# Balancing ecosystem function, services and disservices resulting from expanding goose populations

**DOI:** 10.1007/s13280-017-0902-1

**Published:** 2017-02-18

**Authors:** Ralph Buij, Theodorus C. P. Melman, Maarten J. J. E. Loonen, Anthony D. Fox

**Affiliations:** 1 Team Animal Ecology, Wageningen University and Research, Wageningen, Netherlands; 2 Arctic Centre, University of Groningen, P.O. Box 716, 9700 AS Groningen, Netherlands; 3 Department of Bioscience, Aarhus University, Kalø, Grenåvej 14, 8410 Rønde, Denmark

**Keywords:** Ecosystem functions, Ecosystem services, Goose overabundance, Herbivores, Species interactions

## Abstract

As goose populations increase in abundance, their influence on ecological processes is increasing. We review the evidence for key ecological functions of wild goose populations in Eurasia and North America, including aquatic invertebrate and plant propagule transport, nutrient deposition in terrestrial and aquatic ecosystems, the influence of goose populations on vegetation biomass, carbon storage and methane emission, species diversity and disease transmission. To estimate the implications of their growing abundance for humans, we explore how these functions contribute to the provision of ecosystem services and disservices. We assess the weight, extent and trends among such impacts, as well as the balance of their value to society. We examine key unresolved issues to enable a more balanced assessment of the economic costs or benefits of migratory geese along their flyways, including the spatial and temporal variation in services and their contrasting value to different user groups. Many ecological functions of geese are concluded to provide neither services nor disservices and, ecosystem disservices currently appear to outweigh services, although this varies between regions. We consider an improved quantification of ecosystem services and disservices, and how these vary along population flyways with respect to variation in valuing certain cultural services, and under different management scenarios aimed at reducing their disservices, essential for a more balanced management of goose populations.

## Introduction

In recent decades, goose populations have dramatically increased in most, but not all, populations in the Western Palearctic (Fox et al. 2010) and Nearctic (U.S. Fish and Wildlife Service 2015), mostly facilitated by human-induced changes at the traditional wintering grounds. Demographic evidence suggests that geese benefit from the shift from traditional wetland and low intensity farmland habitats to intensive agriculture (van Eerden et al. 1996; Abraham et al. 2005; Fox et al. 2005) and have escaped population limitation by hunting (Fox 2003). Both factors have also enabled the colonization of new habitats for reproduction which were not available earlier (Fenger et al. 2016); indeed several migratory goose species have become sedentary populations in former wintering areas (Feige et al. 2008). In general, expansions in breeding and wintering ranges have made geese more numerous in many areas, focussing attention on their impacts, most notably the loss of agricultural revenue and the threat to flight safety associated with their abundance. In contrast, assessments of the benefits people derive from geese, resulting from “ecosystem services”, have been limited (e.g. Green and Elmberg 2014) and are hardly ever balanced against the adverse impacts that geese are considered to have (their “disservices” to people).

In this review, we assess the ecosystem services and disservices provided by wild goose populations to human societies. The ecosystem services concept aims to draw attention to the benefits of nature to mankind and, on this basis, achieve a more sustainable use of natural resources and a more equitable distribution of these benefits (MEA 2005). Identifying, quantifying, valuing and monetizing of the ecosystem services are important mechanisms to provide a basis for more balanced decision-making concerning natural resources (Wallace 2007; TEEB 2010). The first step towards a comprehensive assessment of ecosystem services involves the unravelling of ecological complexity (structures and processes) into a more limited number of ecosystem functions (De Groot et al. 2002). These functions, in turn, provide the services that are valued by humans. In our review, this distinction between benefits, ecosystem services and ecological functions is important, especially to prevent double counting (Wallace 2007; Boyd and Banzhaf 2007). The existing literature presents several definitions (e.g. De Groot et al. 2002; MEA 2005; Wallace 2007; Seppelt et al. 2011), but we follow Boyd and Banzhaf (2007) as closely as possible, using their definition that: “ecosystem services are components of nature, directly enjoyed, consumed, or used to yield human well-being”. They make a clear distinction between services and benefits, the latter of which they consider to be the effect of the services. In the same vein, if the benefits are adverse, they originate from ecosystem disservices. In their view, recreation is a benefit, originating from e.g. a configuration of plant species in a landscape which is the ecosystem service. We differ from previous assessments (e.g. Green and Elmberg 2014), which included a range of potential, *indirect* benefits to humankind (such as biodiversity regulation) as ecosystem services. We restrict services or disservices to those functions of geese that *directly* impact humans. In other words, ecosystem services are the ‘end-products’ consumed by human kind as benefits or disadvantages, whereas ecological functions are the underlying processes and intermediate products, that do not necessarily directly benefit or cause disadvantage to humankind (e.g. Boyd and Banzhaf 2007). As stated, this distinction is not always clear and remains the subject of discussion (Wallace 2007; Fisher et al. 2009; Seppelt et al. 2011).

For this reason, we structured this review using the following steps: (a) what are the main ecological functions in which geese play a vital role, i.e. how do goose populations influence their environment? (b) What are the consequences for the environment (effects, intermediate products)? (c) What are the ecosystem services or disservices following from these ecological functions, i.e. which aspects of the ecological functioning of geese are beneficial or detrimental, to humans? Although East Asian goose populations show less favourable conservation status (Jia et al. 2016), we know far less about their ecosystem function, services and disservices, which therefore will not be considered here.

We subsequently assess the weights and trends of the impacts of ecosystem services or disservices and review the balance of their perceived value to society. This can only partly be achieved through a financial assessment of these services. The sense and non-sense of the strict application of financial costs to the validation have been discussed in depth elsewhere (e.g. Farber et al. 2002; Howarth and Farber 2002). Since financial considerations play an important part in societal and political decisions, such a financial assessment may facilitate a more balanced policy making by quantifying benefits and disadvantages. Monetary value is particularly easy to use to assess provisioning services and we review the economic impacts of such services and disservices where possible. For regulating and cultural services, monetizing is more complicated, since the market for these is not well developed (Farber et al. 2002; Sijtsma et al. 2013) and several regulating services in fact represent functions (e.g. pollination) or benefits (e.g. aesthetic values) (Boyd and Banzhaf 2007).

## Ecological functions of geese

### Carriers of other organisms or their propagules

#### Plant and animal dispersal

Bird-mediated passive transport of propagules of aquatic invertebrates and plants is likely a significant means of dispersal for many species, at least locally, especially involving aquatic birds (Figuerola and Green 2002; Green and Elmberg 2014). Such transport may be either by ectozoochory (by adhesion to the outside of animals) or endozoochory (through ingested propagules, requiring mechanisms to survive digestive processes in the alimentary canal of their dispersers; Figuerola and Green 2002). Compared to the rich and diverse literature on ducks as dispersal agents of plant and animal propagules, relatively few studies have addressed the importance of geese in this regard (Green and Elmberg 2014).

On the winter quarters, out of 24 shot brent *Branta bernicla* from a New Jersey saltmarsh, 18 carried seeds of five grass species and three forbs (plus two other unidentified graminoid seeds) on their feet or feathers, all but one of which had potentially adhesive structures to facilitate attachment (Vivian-Smith and Stiles 1994). A study of lower saltmarsh endozoochorous seed dispersal by brent geese showed seeds dispersed through the guts of geese were two orders of magnitude less likely to germinate compared to undigested seeds dispersed by the tide (Chang et al. 2005).

On breeding areas, small-scale propagule dispersal was common in barnacle goose *Branta leucopsis* faeces in Svalbard, mainly grasses and Cyperacean species, but also forbs (especially Arctic Bistort *Bistorta vivipara*) and berries (Bruun et al. 2008). Berries are a major feature of goose diets, especially during post-breeding and pre-migration fattening periods in the Arctic and sub-Arctic, and this may contribute to seed dispersal for such species (Kear 1966). Although most geese evacuate the contents of their intestines before or early into long-distance flight (Klein et al. 2008), and long-distance dispersal events are likely to be rare for this and other reasons (cf. Clausen et al. 2002), experimental studies show retention of seeds and other propagules for longer periods, especially large plant seeds, potentially providing transport of alien and native plant seeds over distances in excess of 1000 km (García-Álvarez et al. 2015). In this way, geese may potentially have contributed to the dispersal of water plants, for example as claimed from temperate areas to Greenland (Bennike and Anderson 1998).

Geese may disperse noxious or toxic weeds that cause problems for agriculture, although a study of resident Canada geese *Branta canadensis* droppings in suburban and urban North Carolina, U.S., found them to be relatively poor vectors of viable seeds: only four plants (3.1%) germinated out of 127 droppings planted (Ayers et al. 2010). Nevertheless, geese retain the potential to disperse alien species (e.g. Best and Arcese 2009; Isaac-Renton et al. 2011; Green 2016).

As well as plant propagules, geese are likely important dispersers of invertebrates. For example, greylag geese *Anser anser* disperse bryozoans (Figuerola et al. 2004), Canada geese are thought to be major vectors of zooplankton in the arctic (Haileselasie et al. 2016), while Louette and De Meester (2004) propose geese as important vectors of zooplankton between Belgian ponds.

#### Spread of disease

Migratory geese cross national borders annually, exploiting a variety of sites where they stop for longer or shorter periods, in the process disseminating a range of pathogens harmful to humans and poultry, including avian influenza, Newcastle disease virus, avian pneumovirus, duck plague virus, and egg drop syndrome virus (Hubálek 2004; Dhama et al. 2008). Some of these, such as avian influenza, have led to major economic losses. Bar-headed *Anser indicus* and greater white-fronted geese *Anser albifrons* are considered the principal reservoir for most of the avian influenza subtypes (Alexander 2000), although the majority of these were low pathogenic forms (Dhama et al. 2008). However, geospatial analysis shows that the Asian distribution of highly pathogenic H5N1 influenza virus outbreaks in domestic poultry was associated with free grazing geese in the region (Gilbert et al. 2006). Migratory bar-headed geese were suggested to act as long-distance carriers of the H5N1 strain in Asia (Chen et al. 2005), based on the genetic relatedness of H5N1 virus isolated from geese in Tibet and Qinghai Lake in China (Prosser et al. 2011). Geese may also be carriers of other diseases that impact birds; for example, histopathological lesions consistent with proventricular dilation disease (PDD) caused by avian bornavirus that leads to high mortality in parrots have been identified in wild Canada geese (Daoust et al. 1991).

In addition to viruses, numerous studies over the past 15 years have shown that Canada goose faeces contain pathogenic protozoa and bacteria (Gorham and Lee 2015). Consequently, Canada geese may pose important health problems at lakes used by people. Canada geese were the dominant source of *Escherichia coli* (44.7–73.7% of the total sources) in four watersheds in the U.S. (Somarelli et al. 2007) and more than 95% of *E. coli* isolates from Canada geese were resistant to a range of antibiotics apart from bacitracin or ciprofloxacin (Fallacara et al. 2001; Cole et al. 2005; Middleton and Ambrose 2005). A single Canada goose can excrete up to 107 faecal coliforms daily, with 3.6 × 10^4^ faecal coliforms per gram of faeces, although only 9% of those were enterotoxin-producing *E. coli* and no *Salmonella* spp. were detected (Hussong et al. 1979). Canada geese have also been linked to water contamination through dissemination of infectious *Cryptosporidium parvum* oocytes (Graczyk et al. 1997; Fallacara et al. 2004) or *Campylobacter* (Rutledge et al. 2013). Campylobacters are among the most significant causes of human gastrointestinal infections worldwide, and the role that waterfowl have in the spread of disease is only now beginning to emerge. Colles et al. (2008) found that many wild geese carry *Campylobacter*, although the highly host-specific genotypes of *C. jejuni* isolated from geese indicate they are unlikely to be the source of human disease outbreaks. Barnacle geese are also a potential vector of toxoplasmosis into a high arctic ecosystem, where the common intermediate host is not present, but Arctic foxes *Alopex lagopus* have suffered infection (Prestrud et al. 2007).

### Defecation

#### Soluble N as fertilizer and fodder

Geese can produce between 58 g day^−1^ (barnacle goose) and 175 g day^−1^ faecal material (Canada goose, c. 2–4% of their body mass; Kear 1963), depositing up to 0.3 droppings m^−2^ day^−1^ in heavily grazing areas (Groot Bruinderink 1989). In wet soils and those with low levels of mobilized soluble nitrogen (N), plant growth may be limited by N. The white deposits on goose faeces contain soluble N in the form of uric acid and ammonium ions, which may enhance plant growth under N limited conditions. This may particularly be the case in Arctic graminoid systems, where limited edaphic N, and short growing seasons constrain spring growth of grass and sedge species eaten by lesser snow geese *Chen caerulescens caerulescens* (Cargill and Jefferies 1984a, b; Bazely and Jefferies 1989; Ruess et al. 1989; Beaulieu et al. 1996). In sub-Arctic Alaskan spring barley *Hordeum vulgare* fields, goose faeces provided more N to the soil and subsequent crop than was generally available, contributing N during the critical early growth phase (Cochran et al. 2000).

This may not be the case further south on staging and wintering areas of geese. Generally, the literature reports almost no winter fertilizing effects from droppings in stimulating grass and cereal growth (e.g. geese feeding on grass and winter cereals; Abdul Jalil and Patterson 1989; Groot Bruinderink 1989). In contrast to Arctic studies, goose faeces added to clipping experiments in north-western Europe showed very little fertilizing effect, presumably because such contributions of N (1–2 kg N ha^−1^, e.g. Rutschke and Schiele 1978) were trivial compared to agricultural fertilizer applications in such situations (100–200 kg N ha^−1^ for intensive cereal production, e.g. Jensen and Schjoerring 2011). However, van den Wyngaert et al. (2001) showed elevated releases of N and phosphorus (P) from above-ground plant material in grazed *versus* ungrazed semi-natural temperate grasslands. They interpreted this potential “fertilizing effect” to rapid leaching of soluble forms of both elements from goose faeces, although effects were short term, confined to the period when geese were physically present. Rye-grass N content in swards grazed by greater white-fronted geese in winter were significantly higher on grazed *versus* ungrazed sites; inorganic soil N followed a similar trend (Shimada and Mizota 2009). These authors concluded goose droppings contributed to elevated levels of inorganic soil N and contributed to grass regeneration.

Several authors have reported on the “fouling” effects of goose droppings, inhibiting vegetation use by other herbivores (e.g. Balkenhol et al. 1984), but hares were equally willing to visit fouled or dropping-free plots in salt-marshes (van der Wal et al. 1998). Because of the combination of highly selective foraging and low levels of digestion of their plant food compared to ruminants, goose droppings can be relatively nutritionally attractive to other herbivores. Hence, sheep and cattle have been observed in spring eating barnacle goose faeces on the Scottish islands of Coll and Gunna (Ingram 1933), while Svalbard reindeer *Rangifer tarandus platyrhynchos* consume barnacle goose droppings because eating grass-rich goose faeces elevated their own food intake rates above normal grazing (van der Wal and Loonen 1998).

#### Contamination of freshwater and urban areas

Geese frequently forage extensively in highly fertilized agricultural habitats, but congregate to densely roost at night on lakes and wetlands, where their excreta represent an external nutrient source of N and P potentially equivalent to contributions from surface water flow (the largest single input source for most wetlands, Manny et al. 1994; Post et al. 1998; Dessborn et al. 2016). During stop-over or wintering periods varying from 2 to 18 weeks, geese (greater white-fronted, bean *Anser fabalis,* Canada, lesser snow, greater snow *Chen caerulescens atlantica* and Ross’ geese *Chen rossii*) added 88–92% (Rönicke et al. 2008), 75% (Post et al. 1998; Kitchell et al. 1999), 85–93% (Olson et al. 2005), and 70% (Manny et al. 1975, 1994) of the P input from all sources to lakes, wetlands and reservoirs in the U.S. and Germany. In addition, geese supplied between 27 and 44% of all N (Manny et al. 1975, 1994; Post et al. 1998; Kitchell et al. 1999; Olson et al. 2005). One modelling framework (taking into account goose foraging behaviour, energy requirements, metabolic constraints and nutrient concentrations in food) estimated a mean annual allochthonous nutrient contribution by herbivorous waterbirds to Dutch freshwater bodies of 382.8 ± 167.1 tonnes N year^−1^ and 34.7 ± 2.3 tonnes P year^−1^, which corresponded to annual surface-water loadings of 1.07 kg N ha^−1^ and 0.10 kg P ha^−1^ (46% of which by greater white-fronted and greylag geese; Hahn et al. 2008).

Such nutrient contributions by geese to aquatic systems may reduce water quality (e.g. Manny et al. 1994; Olson et al. 2005; but see Pettigrew et al. 1997) through adverse increases in phytoplankton, including nitrogen-fixing cyanobacteria and algae (Kadlec 1979; Kitchell et al. 1999; Nürnberg and LaZerte 2016) and create conditions suitable for avian cholera and type C botulism outbreaks (Enright 1971; Wobeser 1981). However, N and P contributions to ultra-oligotrophic shallow tundra ponds from barnacle and pink-footed geese *Anser brachyrhynchus* had little impact on phytoplankton biomass on Svalbard because high biomass of the efficient zooplankton grazer *Daphnia* in the absence of fish and invertebrate predators limited algal growth (van Geest et al. 2007).

In addition to contamination of water sources (e.g. Rutledge et al. 2013), urban contamination by growing urban geese populations is increasing, notably not only in city parks but also elsewhere, enhancing the risk of infections by elevated proximity of geese to humans and livelihoods (Beston et al. 2014; van der Jeugd and Kwak 2017).

### Above-ground grazing and grubbing for subterranean roots and rhizomes

Most monocotyledonous plants show compensatory regrowth to defoliation after biomass removal by grazers, to a greater or lesser extent where nutrients are not limiting (McNaughton et al. 1983; Ferraro and Oesterheld 2002). McNaughton’s (1979) grazing optimization hypothesis predicts that plant production is stimulated at intermediate levels of grazing, whereby goose grazing enhances net primary production and may elevate protein content (Prins et al. 1980; Ydenberg and Prins 1981), confirmed by manipulative studies at the plot (Cargill and Jefferies 1984b) or plant level (Hik et al. 1991; Fox et al. 1998; Fox and Kahlert 2003). Captive barnacle geese grazing on red fescue *Festuca rubra* swards in the Dutch Wadden Sea increased axillary tiller production at grazing levels similar to natural situations (van der Graaf et al. 2005). These findings suggest that grazing geese may at least modestly increase the carrying capacity of monocotyledonous swards, although other studies have failed to find such compensatory growth (e.g. wintering barnacle geese grazing rye-grass-dominated pastures in Scotland; Cope et al. 2003). Such results contrast those of studies where geese consumed plant storage organs, which almost inevitably reduces primary production (e.g. Bélanger and Bédard 1994; Amat 1995).

The longer term effects of grazing may be adverse especially under increasingly intensified grazing by growing goose populations in sensitive Arctic systems. Nutrient levels and a short growing season constrain primary production in Arctic regions, where goose grazing may reduce production of graminoids in comparison to areas where geese were excluded (Gauthier et al. 2004). In Arctic coastal salt marshes, moderate goose grazing on *Puccinellia phryganodes* enhances plant production, but intensified grazing in combination with grubbing for subsurface rhizomes beyond a certain threshold can destroy plant cover, leading to soil erosion and inhibiting plant revegetation over extended periods due to elevated soil surface salinity (Jefferies 1988). Along Hudson Bay coasts, Canada, this process has spread inland to cause further loss of plant cover over large expanses of the Hudson Bay lowlands (Iacobelli and Jefferies 1991; Jano et al. 1998), loss of soil N retention (Buckeridge and Jefferies 2007) and ultimately a runaway trophic cascade analogous to desertification (Williams et al. 1993; Srivastava and Jefferies 1996). In the face of equally rapid increases in goose densities, Arctic freshwater wet meadows show less corresponding declines in plant productivity, although in such systems, grazing may favour mosses over graminoids because of their enhanced ability to access N released from goose faeces near the soil surface (Gauthier et al. 2006). However, in Svalbard, wet habitats appear highly susceptible to vegetation loss, substrate disruption and soil loss as a result of goose grubbing there (Speed et al. 2009); an effect which is increasing with population increase and expansion on the summering areas (Pedersen et al. 2013).

#### Crop loss

Many goose species have shifted from traditional sources of food in natural ecosystems to forage in similar ways in agricultural landscapes, where dense sown single-species crops (such as rotational grassland, early-growth cereals and root crops) and spilled grain offer vastly elevated energetic and nutritional intake rates of food of higher quality compared to that available from natural or semi-natural vegetation types (Fox et al. 2016). The movement from natural ecosystems to farmland habitats has been widespread (Abraham et al. 2005; Fox et al. 2005), suggesting that temperate agriculture has been highly effective at extending the effective carrying capacity of wintering goose numbers (van Eerden et al. 1996). Indeed, changes in feeding habits have potentially supported the growth of populations (Fox et al. 2005). Damage and yield loss to valuable crops by rapid increases in abundance of migratory geese populations have created increasing conflicts over greater geographical areas than ever before (Fox et al. 2016). Studies show that it is difficult and expensive to assess the precise impacts of goose foraging on yield loss (for the purposes of structuring financial compensation), because of other sources of variation (e.g. timing of grazing or timing of harvest). Although at the country level, yield losses are often trivial, individual farmers in areas of greatest goose concentrations suffer disproportionately, necessitating improved solutions to conflict as highlighted elsewhere in this volume. In 2009, some US$21 million were paid in different agricultural subsidies via the national scheme to accommodate geese on farms in Scotland alone, ignoring losses to farmers forgone outside of these schemes (Bainbridge 2017). With increasing numbers and range, such expenditure continues to rise. For example, goose damage and compensation scheme payments in the Netherlands amounted to US$6.4 million in 2000 but had risen to 15.9 million in 2007 and continue to increase to the present (Koffijberg et al. 2017). These increases in costs were due to an increase in goose numbers, in addition to a rise in crop prices, and implementation of new policies (Melman et al. 2009).

#### CO_2_ and CH_4_ emissions

Through their grubbing and grazing, geese can stimulate greenhouse gas emissions such as CO_2_ and CH_4_, especially where geese occur at high densities in temperate and Arctic habitats. About 30% of the annual global emissions of CH_4_—a potent greenhouse gas 28 times more effective at absorbing infrared radiation than CO_2_ (Myhre et al. 2013)—to the atmosphere come from natural wetlands. Intact helophytes conduct CH_4_ produced by methanogenic microbes under anoxic conditions in the soil to the atmosphere by active transport or diffusion (Laanbroek 2010). After having been grazed by greylag geese, emergent *Phragmites australis* shoots emit CH_4_ into the atmosphere much more rapidly relative to the slow diffusion through the stem base in intact plants, with up to five times more CH_4_ released from grazed compared to ungrazed vegetation (Dingemans et al. 2011).

Arctic-breeding geese can reduce both carbon (C) stocks and C sinks in wet tundra through belowground herbivory, which reduces moss and vascular plant photosynthetic tissue (van der Wal et al. 2007). Such grubbing opens up the vegetation mat, exposing the active organic layer to erosion by fluvial outwash, flooding and wind and loss of stored C. As wet tundra provides the strongest C sink function (Sjögersten et al. 2006), the negative impact of geese on the ability of Arctic tundra to sequester C is likely to be disproportional to their overall occurrence. High grazing levels also reduced vascular biomass and litter C pools at two high Arctic habitats, mesic heath and wet meadow and increased decomposition rates at the mesic site, while intermediate grazing increased C storage (Sjögersten et al. 2012). In contrast to Arctic breeding sites, it remains uncertain whether increased populations of Western Palaearctic geese reduce the CO_2_ uptake and thus carbon sink strength of the temperate grasslands from their winter habitat, although goose grazing may substantially impact the CO_2_ fluxes of temperate grasslands (Fivez et al. 2014).

#### Impact on other species

Geese can influence (beneficially or detrimentally) the abundance and diversity of a range of species through their grazing, grubbing and trampling. Persistent goose grazing maintains extremely short uniform grass swards compared to grazing by stock or mammal grazers, which has substantial effects on physiography, structure and physical features of the sward for other organisms present. Socially foraging brent geese rapidly deplete preferred *Festuca* and *Puccinellia* salt-marsh sites in spring and can evict mammalian herbivores such as brown hares *Lepus europaeus* to alternative, less favourable foraging sites (van der Wal et al. 1998; Stahl et al. 2006). The recovery of the population of Aleutian cackling geese *Branta hutchinsii leucopareia* is thought to have led to soil erosion and burrow collapse in a seabird colony in California, where the geese stage in spring (Mini et al. 2013). Grazing by resident Canada geese in tidal freshwater and saltmarshes in the U.S. and Canada affected the food supply, breeding and wintering habitat of a variety of invertebrate and bird species (Haramis and Kearns 2007; Dawe et al. 2011; Nichols 2014). Habitat destruction in the La Pérouse Bay ecosystem by lesser snow geese reduced the local abundance of passerine species such as savannah sparrows *Passerculus sandwichensis* and of shorebirds such as semipalmated sandpipers *Calidris pusilla* (Abraham and Jefferies 1997; Hitchcock and Gratto-Trevor 1997; Rockwell et al. 2003), up to 10 km from the nearest goose colony (Hines et al. 2010). Conversely, moulting greylag geese affected the structure of permanently inundated reed *P. australis* stands (Loonen et al. 1991), favouring the development of feeding habitat for bearded reedling *Panurus biarmicus* and other marshland birds (Beemster et al. 2010). Goose grazing is likely to alter the suitability of nesting habitat for wader populations (Smart et al. 2006), although comparative assessments of breeding wader densities on fields grazed or not grazed by geese may be confounded by other factors (Vickery et al. 1997). Breeding wader populations in the Netherlands showed more positive trends in sites with higher densities of wintering geese than at sites with lower goose densities (Kleijn et al. 2009).

Apart from specific biotic effects, such as loss of cover and food for herbivorous vertebrates and invertebrates, goose grazing changes the physical environment, reducing variance in humidity and temperature and affecting associated biodiversity (e.g. Ford et al. 2013). Reductions in flowering propensity and loss of flowering species impact invertebrate flower visitors and species dependent on pollen/nectar (Meyer et al. 1995), while reductions in plant architecture and structural diversity reduce species richness, abundance and diversity (Sherfy and Kirkpatrick 2003). Geese foraging in wetlands can strongly reduce riparian vegetation diversity over a range of environmental conditions (Sarneel et al. 2014). In temperate brackish marshes, greater snow geese heavily grub the rhizomes of *Scirpus pungens* which alters plant species composition, and influences marsh dynamics by enlarging ice-made depressions which are colonized by other species (Gauthier et al. 2006). On islands without Arctic foxes, Aleutian cackling geese have fundamental effects on the terrestrial plant community and structure and ecosystem dynamics (Maron et al. 2006). A study on offshore islands in Canada showed an invasive alien goose species (a large-bodied subspecies of Canada goose native to the central prairies of North America) fed selectively on exotic introduced grasses and avoided native forbs (Best and Arcese 2009; Isaac-Renton et al. 2011), facilitating both the local increase and the spatial spread of exotic grasses. In the extreme, trophic cascades initiated by goose grazing (described above) from La Pérouse Bay have denuded previously vegetated areas and exposed saline organic-rich substrates and reduced invertebrate communities, particularly midge, spider and beetle communities (Milakovic et al. 2001; Milakovic and Jefferies 2003). In contrast, Bruun et al. (2008) showed that endozoochorous goose propagule dispersal in the Arctic can potentially generate and maintain local plant species richness, as well as enabling long-distance dispersal and range shifts in response to climate change.

### Conversion of plant biomass to live tissue

Through their growth and reproduction, wild geese convert plant material into meat, thus providing an importance source of fat, protein and other consumptive products for humans and other organisms. Wild geese are important food for Inuit people in northern Canada and throughout the polar region (Lévesque and Collins 1999; Krcmar et al. 2010) as well as to hunters and consumers of wild goose meat at more southerly latitudes. The eggs of geese may still be an important source of protein to indigenous peoples (MacMillan and Leader-Williams 2008), while goose down and feathers were formerly used for decoration of bows and arrows (Ashwell 1978), bedding, and insulation (although farmed geese have largely taken over this supply, MacMillan and Leader-Williams 2008). Greenland Inuit use goose bones to make small sewing needles (Damas 1984). In addition to providing resources to people, geese are a major food source for eagles (McWilliams et al. 1994), Arctic foxes (Bantle and Alisauskas 1998), polar bears *Ursus maritimus* (Gormezano and Rockwell 2013; Prop et al. 2015), and wolves *Canis lupus* (Wiebe et al. 2009). Breeding colonies of geese may help sustain predator communities even in their absence, such as Arctic foxes surviving on cached eggs of Ross’s and lesser snow goose during winter (Samelius et al. 2007). Geese can also influence the local abundance of other vertebrates in other ways: nesting geese often vigorously defend their nest and its immediate surroundings against potential predators, thus providing refuges for other taxa in the vicinity (e.g. Giroux 1981; Allard and Gilchrist 2002).

## Ecosystem services and disservices by wild geese populations

In the face of growing goose populations, it is important to understand how the ecological functions of geese populations result in ecosystem services. We therefore focus on the benefits and disadvantages originating from the ecological functions, i.e. those aspects of ecological functioning of geese beneficial or detrimental, respectively, to humans. In reviewing the ecosystem services by geese we follow the United Nations Millennium Ecosystem Assessment (MEA 2005), by classifying them according to their type as provisioning, cultural, regulating, and supporting services. While reviewing the ecosystem services of a group of species, it is important to use a clear definition. The essential basis for all types of ecosystem services is the relationship with man (beneficial or detrimental). The absence of such a relationship infers a process and not an ecosystem service or disservice (Goulder and Kennedy 2011; Tallis and Polasky 2011; Boyd and Banzhaf 2007). For ecologists familiar with the fundamental meaning of ecological processes, it is tempting to interpret ecological functions as ecosystem services, e.g. including effects on other taxa (cf. Green and Elmberg 2014). Here we limit ourselves to recognized ecosystem services that directly impact humans, aware that, with increasing knowledge, some ecological processes might be eventually become acknowledged as ecosystem services.

### Provisioning services

Provisioning services refer to the production of vegetable and animal foods by relatively “natural” ecosystems (MEA 2005), as well as of production systems in which man plays a role, such as intensive farming systems. These services include the consumptive use of geese, for products such as meat, eggs, down, and feathers. For example, the annual economic value of the waterfowl subsistence harvest to several thousand Inuit varied between US$66 000 and US$150 000 in 1988–1997 (Krcmar et al. 2010). Canada geese killed during the Native Harvest in the Hudson Bay Lowland of Ontario contributed 120 000 kg and lesser snow geese 88 000 kg of edible biomass per annum (Berkes et al. 1994), equivalent to US$6–US$8.5 per kg of edible poultry meat in settlements in 1990.

There is also a disservice in this category. The main provisioning disservice of geese is crop yield loss as a result of their foraging on agricultural fields, which much exceeds the monetary value of the provisioning services. Such yield losses have strongly increased and continue to rise in Europe (MacMillan et al. 2004; Bjerke et al. 2014; Bainbridge 2017; Koffijberg et al. 2017) and in North America (e.g. Radtke and Dieter 2011). In the Netherlands, the damage to food production is estimated at US$10.6–21.2 million per annum (Melman et al. 2011; Guldemond and Melman 2016).

### Cultural services

Cultural services are the “nonmaterial benefits people obtain from ecosystems through spiritual enrichment, cognitive development, reflection, recreation, and aesthetic experiences” (MEA 2005), which for geese may relate to recreational hunting, birdwatching and ecotourism, but also science and education. Recreational goose hunting differs from subsistence hunting because of the emphasis on enjoyment of the activity by hunters, rather than on the product obtained (which falls under provisional services). Recreational goose hunting makes an important contribution to local, state and national economies in the U.S., where the Fish and Wildlife Service maintains millions of square kilometres as National Wildlife Refuges open to public hunting. In 2006, waterfowl hunters represented 10% of all hunters in the U.S., 7% of all hunting-related expenditure, and 6% of all hunting equipment expenditure (Carver 2008). It is estimated that 1.3 million waterfowl hunters (including 700 000 goose hunters) spent an estimated US$900 million on waterfowl hunting trips (including food, lodging, transport, equipment) in the U.S. in 2006 (Carver 2008). Waterfowl hunting expenditures in 2006 created 27 618 jobs and US$884 million in employment income, strongly boosting local economies. Revenue from waterfowl hunting (although it is unclear what proportion were goose or duck hunters) totalled c. US$87 million (in 2009) for the 2005–2006 hunting season in the state of Mississippi alone, supporting 512 full- and part-time jobs in six counties (Grado et al. 2011). Waterfowl hunting is also important pastime in the E.U., where 7 million hunters shoot at least 7.6 million waterfowl annually (Mooij 2005; Hirschfeld and Heyd 2005). Visitor expenditure by goose hunters in Scotland in 1997–1998 was estimated to be 40% more than the considerable number of birdwatchers watching geese (MacMillan and Leader-Williams 2008).

People may also positively value wild goose populations for birdwatching or simply from the pleasure of knowing they exist (e.g. MacMillan et al. 2004). In general, birding is the fastest-growing outdoor recreational activity in U.S. and the most promising branch of ecotourism in terms of economic impacts, with a high potential to contribute to local communities (Şekercioğlu 2003). Although little quantified, specific non-consumptive interest in geese is increasing the U.S. and Canada and 2–3 day goose festivals geared specifically for greater snow or brent geese attract thousands of visitors, bringing substantial local economic benefit (Hvenegaard and Manaloor 2006; SGSBC 2009; Hvenegaard 2011). The annual revenue from birdwatching and eco-tourism in the four main spring staging areas of greater snow geese in Québec was estimated at c. US$14 million (Bélanger and Lefebvre 2006). Snow goose festival visitors spent an estimated US$73 000 in one local area of western Canada in April 2000 (Hvenegaard and Manaloor 2006), whilst brent festival visitors spent c. US$398 000 in another area in April 2003 (Hvenegaard 2011). Goose-related tourism has been similarly shown to contribute importantly to the local economy in the E.U. (Edgell and Williams 1992).

Both birdwatching and hunting provides an emotional benefit which, by definition, exceeds the money that is invested. To comprehensively assess the benefits of conserving wild geese to society their non-market benefits therefore also need to be estimated, even if they are difficult to quantify in financial terms. A Scottish survey showed that “willingness to pay” for goose conservation on the Scottish island of Islay outweighed costs of damages to agriculture by a factor of 113–700, depending on different population trajectory scenarios for endangered or non-endangered goose species (MacMillan et al. 2004). Farmers will only participate in goose conservation if they receive adequate compensation for losses that accrue to them, necessitating government compensation schemes. Total costs to tax-payers from implementing such a scheme (estimated at c. US$1.2 million/annum in 2008) was entirely justified because the benefits of goose conservation greatly exceeded the costs and were dispersed amongst the general population (MacMillan and Leader-Williams 2008).

Because air travel supports cultural activities such as recreation, we include the collision risks to aviation posed by geese under cultural disservices (see Bradbeer et al. 2017). The most prominent negative impact is the loss of human life resulting from an airplane crash after it collided with geese. Other costs involved include among others those to manage goose numbers around runways (habitat management, goose repellents), goose relocation or culling operations, and airplane damage repair costs. Wildlife strikes costs the U.S. civil aviation industry approximately US$500 million annually in the U.S. (Cleary et al. 2004), and ducks and geese together account for 7% of the strikes but are responsible for 30% of the strikes that cause damage to the aircraft (Federal Aviation Administration 2016).

### Regulating services

Regulating services are the services that ecosystems provide by acting as regulators, e.g. regulating the quality of air, water, soil and climate or by providing food and disease control. In terms of disease regulation and surveillance, geese provide both ecosystem disservices and services. As hosts and vectors for a wide range of pathogens, including those transmitted to poultry or humans (Hubálek 2004; Olsen et al. 2006), geese provide an ideal basis for disease surveillance. In particular, certain subtypes of influenza A viruses have been detected in white-fronted, barnacle, greylag, brent, bean, and pink-footed geese, making them useful study species for monitoring temporal variation in avian influenza prevalence in order to predict and prevent economic losses to the poultry industry and also epidemics or pandemics in humans (e.g. Hoye et al. 2010).

Among the regulating disservices associated with increasing goose abundance are urban pollution, eutrophication of freshwater sources, methane efflux, loss of plant cover, soil erosion, and loss of carbon storage. Their impacts on the economy are hard to quantify; however, the relative impact of these regulatory disservices is rather limited compared to other factors that cause climate change, soil erosion or pollution. For example, the contribution to climate change from loss of C storage following grubbing by Arctic geese is likely to be very limited compared to impacts of thawing permafrost or wildfires (e.g. Schuur et al. 2008; Mack et al. 2011), while methane efflux following grazing of wetlands is probably negligible compared to the impact of anthropogenic non-CO2 greenhouse gas emission (e.g. Montzka et al. 2011). Although locally, urban and water pollution by geese may cause significant human discomfort, globally it constitutes merely a fraction of the pollution with sediment, nutrients, bacteria, oil, metals, chemicals, road salt, pet droppings and litter from the numerous contaminant emitting sources in urban areas.

### Supporting services

This category includes services that are “necessary for the production of all other ecosystem services” (MEA 2005). Ecological functions of geese discussed above, such as plant or animal dispersal, nutrient cycling, influencing primary production and species diversity, are frequently classified as supporting services (or disservices in the case of their adverse effects). Most refer to ecological processes which do not directly impact humans and do not therefore constitute ecosystem services. Long-distance goose dispersal of seeds may influence plant communities at large spatial scales, but do not involve species providing valuable fruits or timber directly to human societies, so under these circumstances fail to meet service/disservice criteria. However, by enabling plant and animal communities to shift their distributions to adapt, for instance to climate change, these functions are likely to support the development of healthy and adaptive aquatic systems in the future, which in themselves may increase C sequestration by maintaining communities adapted to local climate. In contrast, there are very few indications that nutrient cycling by geese influences crop production.

## Balancing services and disservices

The recent expansion of goose populations has generated much debate, emphasizing ecosystem disservices caused by geese, most importantly their influence on aviation safety and economic loss in agricultural sector. A more balanced assessment of ecosystem services and disservices, their weight and trend of impact and societal validation is essential to better inform decision-making with regard to population management. When balancing ecosystem services and disservices, the strict categorization based on the typology of the Millennium Ecosystem Assessment is not entirely satisfactory. For example, supporting services constitute a confusing category because they provide the conditions under which the other services can be achieved, rather than representing services on their own. Because one of the main aims of the ecosystem services concept is to monetize the benefits and disadvantages (Sukhdev 2008), the overlap in services classification complicates any overall valuation of such services and disservices. Some services differ according to perception between societal groups; e.g. goose hunting simultaneously generates both large economic benefits and strong dissatisfaction to other user groups (notably birdwatchers), for which account need to be taken when estimating the relative societal costs/benefits (Table 1). In general, ecosystem services operate at a range of spatial scales, but production per capita is greater at temperate latitudes for most services (Table 1). Also the societal or economic validation, whether positive or negative, is strongest for those services produced mainly at more southerly latitudes. However, because the rate of goose population increase is greater at higher latitudes (Ramo et al. 2015), those services with greater per capita production rates at northern latitudes, such as loss of carbon storage and production of consumer products (meat, down, feathers), are amplified at such latitudes.

**Table 1:** Overview of ecosystem functions and services or disservices provided by wild goose populations. Latitudinal impact per capita indicates whether the contribution per goose to the service or disservice is greater in Arctic/northern latitudes (N) or temperate/southern latitudes (S); societal or economic validation refers to the societal or monetary value assigned to the service or disservice by society as a whole (qualified as follows: −/−−− negative to very negative impact; +/++ positive to moderately positive); and the spatial extent of the impact refers to impacts at local, regional or global spatial scales. Type of service refers to *P* provision, *R* regulating, *S* supporting and *C* cultural services or disservices

Ecosystem function	Associated ecosystem service (+) or disservice (−)	Benefit or disadvantage	Type of service	Main latitudinal impact per capita	Societal or economic valuation	Spatial extent of impact
Defaecation	Soluble N as fertilizer in cultivated areas	Increased crop growth (sub-Arctic spring barley)	R	N	Negligible	Local
	Soluble N as contaminant of drinking water	Diminished quality of potential drinking water	R	S	−	Local/regional
	Additional nutrients for livestock	Increased livestock fodder	R	N/S	Negligible	Local
	Contamination of urban areas	Human discomfort	R	S	−−	Regional/local
Grazing and grubbing	Removal of plant biomass	Crop loss	P	S	−−−	Regional
	Habitat modification	Maintenance or reduction of species diversity^a^	R	N/S	−	Local/regional
	Destruction of plant cover, soil erosion inhibiting plant revegetation	Soil erosion	T or S	N(S)	−	Local
	CH_4_ emission Loss of stored C (wet tundra)	Climate change	R	N/S	−	Global
		Climate change	R	N/S	−	Global
Conversion of plant biomass to live tissue (reproduction, growth)	Production of meat, feathers, other raw materials	Food Sleep comfort (pillows)	P C	N/S	+	Local
	Presence of geese (including ecological performance)	Joy for birders	C	S	++	Regional
		Revenues for recreational entrepreneurs	C	S	++	Local
		Consumptive use of geese for hunting	C or P	N/S	++	Regional
		Development of scientific theory, output and education	C	S	+	Regional
		Risk of collisions with airplanes Human casualties Damage prevention costs Aircraft damage	C	S	−−−	Regional/global
			C	S	−−−	Regional/global
			C	S	−−−	Regional/global
			C	S	−−−	Regional/global
Carrier of other organisms or their propagules	Spread of disease to humans and poultry	Increased incidence of human and livestock disease and death	C	S	−−	Global
	Indicator of spread of pathogens harmful to humans and poultry	Improved disease surveillance	C	S	+	Regional/local
	Deposited seeds, forbs, berries of: useful plant species harmful or noxious plants	Maintenance of plant species diversity^a^	R	S	Negligible	Regional/global
		Decrease of agricultural productivity	P	S	Negligible	Regional/global

^a^Whilst these categories represent no clear direct benefit or disadvantage to humankind and are therefore not considered as resulting from a service or disservice here, maintenance of biological diversity does clearly benefit humankind ecologically and financially at some level

## Discussion

At present, the adverse effects of the strong growth in goose populations on human well-being (ecosystem disservices) appear to be outweighing ecosystem services provided by geese. However, despite the increasing interest in the use of the concept in science and policy-making, many ecosystem services remain difficult to quantify, to evaluate and to monetize, which complicates weighing the costs and benefits of disservices and services (Green and Elmberg 2014), especially when estimating the cultural (information, enjoyment, emotional) value of geese. Several factors contribute to the complexity of assessment. First, it is tempting to interpret ecological functions as ecosystem services based on knowledge of the importance of those functions for ecological systems, but many functions may not constitute services consumed by human society (Tallis and Polasky 2011). Many ecological functions described here might be essential to the ultimate provision of ecosystem services, but valuing these functions as services would lead to double-counting (cf. Boyd and Banzhaf 2007; Fu et al. 2011). The use of different evaluation methods also confounds objective assessment of ecosystem services and disservices, not least because of their values to different sectors of society (e.g. Goulder and Kennedy 2011). Assessments can vary from being descriptive and subjective to being defined in clear economic costs. In this review, a multitude of studies, ranging from ecological descriptions to precise societal impact studies of geese were considered, based on very different methods. These differences hamper a consistent, unequivocal comparison and quantification of the services provided by geese. Overall, the most important ecosystem services contributed by wild geese populations are their provisional services (meat, down, and feathers) and their cultural (information) value, for birdwatching and hunting. Such cultural services can be highly valued by recreational waterfowl hunters and birdwatchers (MacMillan and Leader-Williams 2008), and may also contribute to investments in equipment, hotels and the food service industry.

Some user groups prioritize certain ecosystem services over others, leading to conflict, for example, when the cultural appreciation of ecosystem services and disservices differs between user groups. Examples are landowners with property damage from geese versus the general public enjoying their presence (Coluccy et al. 2001), and conflicts between hunters and birders over the pleasure of geese from hunting or from birdwatching (Adams et al. 1997). Goose shooting (also for damage control) is disapproved of by a majority of people in some E.U. countries with important goose populations (e.g. Jacobs 2007; MacMillan and Leader-Williams 2008). However, hunting and birdwatching may be combined by allowing hunting only on specific days through the winter, or by providing refuges from hunting within a wetland complex, that are also the sites for birdwatchers. Even in case of such spatial or temporal segregation, behavioural changes in geese such as increasing goose weariness of humans due to hunting (Gerdes and Reepmayer 1983; Madsen 1985) may affect the joy from birdwatching. Hunting may have other adverse side-effect, such as the risk of lead poisoning through the ingestion of lead ammunition (Mateo 2009). Choices then need to be made and will differ between localities, preferably based on monetization of the different services; as we have seen, goose shooting may generate more short-term revenue for local economies than birdwatching (MacMillan and Leader-Williams 2008), whereas birdwatching tourism has a greater potential to improve the long-term financial and environmental well-being of local communities (Şekercioğlu 2002, 2003). Both services might be sustained by restricting hunting in space, time or numbers, resulting in sustainable exploitation of a population which can still be observed and enjoyed. Although in many cases, combining consumptive and non-consumptive uses of geese in the same area may appear neither possible nor desirable, addressing the apparently conflicting issues at appropriate spatial or temporal scales can provide innovative solutions. Seen in this light, the concept of ecosystem services may be able to deliver results which can directly support the development of policy. However, choices based on monetization will not be possible in many situations; for example, monetization is very hard to accomplish especially for cultural services which have no real market. In such instances, identification and quantification of ecosystem services can help decision making, but monetization does not deliver a perfect mechanism.

Ecosystem services/disservices differ along migratory goose flyways, such that ecosystem services/disservices and impacts on well-being are subject to spatial and temporal variation. This may be the result of fluctuations in seasonal abundance, climate change or other anthropogenic influences, such as changes in food availability, or a combination of these. Presently there is a disproportionate burden of disservices associated with intensive agriculture on countries or regions within the major wintering and spring staging areas. These include countries in North-west Europe, where nutrient-rich, ‘industrial’ grasslands provide ideal wintering or stopover grounds for Arctic goose populations (Fox et al. 2005; Van der Graaf et al. 2007). Rapid changes in the phenology and abundance of geese at staging and wintering areas result in shifts in patterns of services and disservices. Increasing use of urban areas in parts of Europe and U.S. (e.g. Beston et al. 2014) has rapidly increased disservices due to the pollution of urban parks and water sources with faeces and associated risks of zoonotic disease (Rutledge et al. 2013). Temporal and spatial shifts in wintering and staging areas can be related to rapid adaptation of geese to changes in human hunting pressure and disturbance (Bechet et al. 2004; Klaassen et al. 2006), climate change (Lehikoinen and Jaatinen 2012; Ramo et al. 2015), natural predation pressure (Jonker et al. 2010), habitat alteration (e.g. Prop et al. 1998; Clausen and Madsen 2016), or food availability and exploitation (Arzel et al. 2006). Increased hunting (including derogation shooting or culling to prevent crop loss) has contributed to spatial shifts in services and disservices when geese start to use new, safer areas, but where they become increasingly shy, at the level of local farms, regions to countries. Apart from a spatial redistribution of the service/disservice, hunting can relocate geese to new, previously unoccupied areas, resulting in greater risk to aviation (Sodhi 2002), loss of birdwatching opportunities (Dahlgren and Korschgen 1992), and paradoxically, increasing total crop damage and conflict with agricultural interests (Bélanger and Bédard 1990; Riddington et al. 1996). This illustrates that human efforts to locally reduce ecosystem disservices provided by geese can have adverse side-effects by (1) increasing associated disservices, (2) increasing other disservices, or (3) reducing ecosystem services in the same or other areas. Such problems can probably only be overcome by coordinated management of flyway sub-populations at local and international scales, involving effective representation of all key stakeholder groups within a flyway.

## Conclusions

Many of the ecological functions of geese do not provide services or disservices, because they do not directly benefit or disadvantage humankind. The concept of ecosystem services/disservices is helpful to derive a more balanced overview of those functions of geese that are of value or harmful to humankind. Adverse effects of geese on agricultural crop production, tundra vegetation and aviation have raised concerns about further increases in goose numbers, strengthening in the call for flyway-scale management plans that include culling. For societal decisions, it would be helpful to monetize all ecosystem services provided by geese, but it is acknowledged that not all services can be monetized, especially non-market services such as aesthetic or informational value (Sijtsma et al. 2013). Moreover, the categorization into services and disservices depends upon the societal group concerned. The translation of ecosystem disservices of geese into management plans would also benefit from a stratified structure to deal with migratory behaviour. Many of the disservices are local problems or differ in intensity seasonally as long as the geese migrate away from the area, especially towards a sparsely human-populated Arctic. However, the level of disservice increases when geese become resident during summer or form denser flocks in the intensively used agricultural landscapes of temperate areas (Meire and Kuijken 1991; Van der Jeugd et al. 2009). It is important to understand that geese have adopted these new strategies and patterns as a consequence of human-induced changes in the landscape, crop quality and conservation (Owen et al. 1987; Van Eerden et al. 1996). In other words, humankind has triggered many of the increases in ecosystem disservices caused by geese, whereas efforts to reduce costs by other means than population control through harvest have been limited to date. These include, for example, creating refuges and scaring geese into the refuges, which has not been adequately implemented in some countries with high conflict (e.g. Koffijberg et al. 2017), although they provide a potentially cost-effective alternative to present compensation schemes especially when combined with habitat management (such as reducing goose access to crop leftovers elsewhere; Jensen et al. 2008). We consider that an improved quantification of ecosystem services and disservices along flyways is essential to provide a more balanced assessment of the costs and benefits of migratory geese, and how these vary along population flyways with respect to variation in valuing certain cultural services, and under different management scenarios aimed at reducing their disservices.
